# The Impact of Alternative Foods on Consumers’ Continuance Intention from an Innovation Perspective

**DOI:** 10.3390/foods11081167

**Published:** 2022-04-18

**Authors:** Chun Yang, Xuqi Chen, Jie Sun, Chao Gu

**Affiliations:** 1 School of Design, Jiangnan University, Wuxi 214122, China; yc004009@gmail.com; 2 Department of Agricultural and Resource Economics, University of Tennessee, Knoxville, TN 37996, USA; xchen88@utk.edu; 3 Department of Culture and Arts Management, Honam University, Gwangju 62399, Korea; 20208429@my.honam.ac.kr

**Keywords:** food innovation quality, consumer perception, alternative foods

## Abstract

This paper aims to model consumers’ perceptions and preferences toward alternative foods. We conducted a survey of 519 people and analyzed their responses using a structural equation model. The article discusses the role of food innovation quality (FIQ), a concept developed from innovative design, which shows how consumers perceive the quality of products in an innovative context. Further, the paper discusses the relationship between this concept and promoting consumer acceptance of alternative foods. Studies suggest that higher FIQ may lead to increased consumer satisfaction with alternative foods, which may in turn lead to higher levels of trust and continuation. Moreover, expectations play a significant role in FIQ and in the perceived value of alternative foods in the model. This illustrates that the promotion of alternative foods in an innovative manner should include establishing a practical mechanism for meeting consumer expectations. Given the continued growth in global food demand, it is both effective and beneficial to promote alternative foods through innovative design as part of a broader food industry approach. On the one hand, alternative foods produced in an innovative manner serve to energize the consumer market by expanding dietary choices. On the other hand, alternative foods, which include new forms of meat products, contribute to the alleviation of the problem of meat production capacity in agriculture. In addition, the alternative foods process eliminates the emission of large amounts of carbon dioxide by traditional agriculture, increasing the sustainability of food production.

## 1. Introduction

### 1.1. Research Background and Purpose

In 2050, the global population is expected to reach 9 billion. Therefore, the demand for food is expected to increase by 70 percent [1]. Current estimates indicate that there will be a 40–75% increase in protein consumption in the future [2]. The continued demand for meat-based diets is contributing to the degradation of the environment [3]. Reforming livestock farming is essential for changing the status quo [4]. Additionally, it is necessary to develop new food products that are edible by humans to alleviate the pressure of food shortages on animal husbandry. As a result, agronomists, nutritionists, and food scientists are exploring and developing new alternative proteins as food sources. Unlike traditional animal and plant proteins, alternative proteins are derived from plants, insects, and new technologies utilizing cell culture or fermentation, producing higher nutritional value while causing less environmental harm [5]. Alternative proteins are considered sustainable food alternatives because they differ from existing foods in terms of resources, production processes, and environmental footprints [6]. The successful promotion of alternative foods depends not only on their development by researchers, but also on acceptance by consumers. The market for plant-based meat substitutes is quite mature. McDonald’s and KFC have launched plant-based meat burgers, and consumers are accustomed to consuming plant-based meat alternatives [7]. In addition, consumers are gradually becoming more accepting of insect meat [8]. The majority of consumers of insect meat are wealthy city dwellers, who view this alternative as an innovative concept [9]. Many consumers, however, do not accept insects as a food source [10]. Furthermore, there are many consumers who are afraid to try new foods (alternative foods) and have difficulty accepting them [11].

Innovative food design plays a significant role in solving this issue by developing new products that meet environmental and technological requirements while stimulating consumer desire for consumption through technological innovation [12]. Moreover, we need to change consumers’ first impressions of food through innovative design, including creative restaurants, innovative dining experiences, food shapes and colors, etc. If a consumer is satisfied with his or her experience after completing the initial consumption of a product, he or she is likely to become a trusted user and supporter of the product, with the intention of repeat consumption. In general, consumers who have had alternative food experiences have a positive attitude and perception of alternative foods, and they are inclined to continue eating alternative foods for a long period of time. By understanding the factors that influence the acceptance of alternative foods by such consumers, we may be able to increase the number of such consumers and promote environmental sustainability. As a result, this study will target consumers who have consumed alternative foods. We examine the main factors that influence their acceptance of alternative foods, test their correlation, and then present improvement suggestions for government, industry, consumers, and others.

### 1.2. Alternative Foods

Basically, alternative foods refer to three different types of non-meat dietary proteins: plant proteins, cultured meat proteins, and insect-derived proteins [7]. Aside from the controversial cultured meat [13], plant meat and insect-based foods have a certain degree of acceptance, particularly plant meat [7]. In general, consumers are more interested in eating insect-based foods that have been prepared in creative ways, rather than simply cooking them [14]. Inspect-based foods possess a greater nutritional value than other alternatives, and are more economical to produce [15]. There are many insect-based foods that provide adequate energy and protein, which could meet human nutritional needs, and which are rich in amino acids, monounsaturated fatty acids (MUFA) or polyunsaturated fatty acids (PUFA), and various trace elements such as Cu, Fe, Mg, Mn, P, Se, Zn, B2, B5, B7, and B9.

### 1.3. Research Scope

In this study, the concept of alternative foods is primarily focused on inspect-based foods that are currently available on the market or that are provided in restaurants. As commercial and restaurant alternative foods already have relevant food safety and quality certifications, this study does not address such certification concerns.

## 2. Theoretical Background and the Research Model

### 2.1. Continuance Intention (CI), Trust (TR), and Satisfaction (SAT)

Continuance intention is the action taken by the consumer after experiencing or utilizing a product [16]. It could be used to assess the success of a product or service [17]. In order to foster the acceptance and promotion of alternative foods, it is more important to maintain consumers’ continuance intention or behavior beyond the first purchase or consumption [18].

Understanding consumer behavior in uncertain and risky circumstances requires consideration of trust [19]. In general, trust is regarded as a positive outcome of “perceived probabilities”, “confidence”, or “expectations” [20]. Considering the prevalence of food safety issues [21], trust plays a significant role in consumers’ decision-making [21,22].

Generally, satisfaction refers to the comparison between expectations and the experience of a product or service [23]. In addition, satisfaction is a product of evaluating service and experience. It has been shown to be a major predictor of future behavior [24]. According to some scholars, satisfaction may also increase consumer loyalty [25,26], resulting in increased intentions to reuse a product or service, as well as increased continuance intentions to purchase a product or service [19].

A number of studies have shown that satisfaction has a positive influence on continuance intentions [27,28,29]. In addition, trust is a moderator between satisfaction and continuance intention [30]. In other words, consumer satisfaction will affect consumer confidence in products, brands, or companies [20], thereby determining whether consumers will continue to buy those products in the future.

In line with the above theory, this study believes that once consumers are satisfied with alternative foods, their satisfaction will affect their trust in alternative foods and their future continuance intentions. Therefore, this study assumes that:

**Hypothesis** **1** **(H1).** *Satisfaction is a significant positive associated with consumers’ trust in alternative foods*.

**Hypothesis** **2** **(H2).** *Trust is a significant positive correlated with consumers’ continuance intention to purchase alternative foods*.

**Hypothesis** **3** **(H3).** *Satisfaction is a significant positive associated with consumers’ continuance intention to purchase alternative foods*.

### 2.2. Food Innovation Quality (FIQ)

Innovation in the food industry is a critical component in achieving competitive advantage [12]. Different restaurants and manufacturers use different methods for food innovation. Consumers, however, may have uncertainties regarding food safety [31], ingredients’ source [32], and processing methods for innovative foods.

Research indicates that consumers prefer whole foods [33]. Innovative foods often claim to provide health, safety, or sustainability benefits that consumers cannot clearly verify, which makes it difficult to monitor their effect. As a result, consumers are less likely to continue using such products [34]. Therefore, innovative food design should eliminate consumer anxiety and mistrust, and ultimately gain trust by implementing such measures as transparency in food processing and reducing the distinction between alternative foods and traditional foods. According to the perspective of innovation perception, perceived naturalness [35] and perceived familiarity [36] can both trigger consumers’ perceptions of innovation.

Furthermore, culinary creativity with ingredients has the potential to enhance consumer perception and enthusiasm. Steier et al. developed a system model of haute couture creativity and innovation by establishing a premium for excellent creativity and high quality, ultimately influencing the development of the industry [37].

Food quality may influence consumers’ intentions and behaviors, as it relates to the perceived quality of foods [38]. Some scholars consider food quality to be the most important factor [39]. Food’s innovative quality is interpreted by how the consumer perceives the relative quality of the product within the innovation dimension, and is considered a key predictor of consumer satisfaction [40]. Innovations in food quality lead to increased consumer satisfaction [41]. Some scholars, however, believe that the negative impact of low-quality food on consumer satisfaction is greater than the positive impact of high-quality food [42]. According to this study, consumers will be more satisfied if they perceive the high quality of alternative foods. Thus, this study assumes:

**Hypothesis** **4** **(H4).** *Food innovation quality (FIQ) is a significant positive associated with consumers’ satisfaction in alternative foods*.

### 2.3. Perceived Value (PV)

The definition of value reflects the optimization of capital costs associated with production or acquisition, i.e., providing products and services that are both high-performing (use value) and attractive (premium value) [43]. The perceived value of a product or service is the value in the eyes of consumers. In this regard, perceived benefits and costs could be viewed as a trade-off [44]. It is more likely that consumers will return to companies with higher perceived value [45]. In this study, we classified consumers’ perceived value of alternative foods into non-economic value (nutritional value and environmental value), and economic value. The perception of value increases when consumers recognize the nutritional value of alternative foods and accept their costs. Based on previous studies, perceived value is one of the determinants of satisfaction. Perceived value has a positive impact on satisfaction [46,47]. Furthermore, perceived value is directly related to intent. A positive perception of value will result in a higher level of willingness to buy or use, whereas a negative perception will result in a reduction of such willingness [48]. Thus, this study assumes:

**Hypothesis** **5** **(H5).** *Perceived value is a significant positive associated with consumers’ satisfaction in alternative foods*.

**Hypothesis** **6** **(H6).** *Perceived value is a significant positive associated with consumers’ continuing intention to purchase alternative foods*.

### 2.4. Expectation (EXP)

Consumer expectations are determined by the difference between the expected and the perceived quality of a product [49]. As long as expectations are met, customer satisfaction will increase, resulting in repeated purchases [50]. Karlsen, Madsen, and Baadsgaard (1996) posited that, in order to influence consumers’ choice of food, a time-based horizontal dimension (used to distinguish between pre- and post-purchase quality perceptions (expected quality and experienced quality)) and a vertical dimension that describes purchase intent based on consumers’ perceptions of quality should be considered [51]. Accordingly, we propose that consumers’ expectations of alternative foods influence satisfaction through the quality of technological innovation (horizontal dimension) and the perceived value of alternative foods (vertical dimension), which impact intentions and behaviors. Therefore, the following assumptions are made:

**Hypothesis** **7** **(H7).** *Expectation is a significant positive associated with the food innovation quality of alternative foods*.

**Hypothesis** **8** **(H8).** *Expectation is a significant positive associated with consumers’ perceived value of alternative foods*.

### 2.5. Proposed Theoretical Model

**Figure 1:**
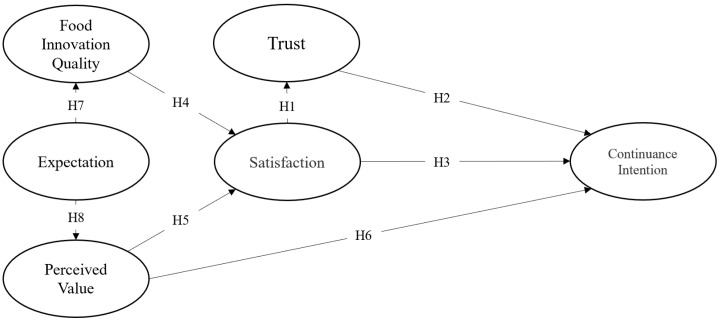
Research structure.

This study proposes the following model (Figure 1) based on the discussion above. Specifically, the theoretical model consists of six constructs, including continuance intention, trust, satisfaction, food innovation quality, perceived value, and expectation, and eight related research hypotheses.

### 2.6. Definition and Measure of Variables

The questionnaire items in this research were designed in light of the research topic and were based on the relevant literature. In Table 1, the operability definition, items, and scales are provided. Given that the subjects of this study were consumers who have eaten alternative foods in the past, this study required them to recall their first experience with alternative foods, when asked about their expectations. Therefore, the expectation construct included a premise—“Please recall the moment when you first planned to consume an alternative food...”—to remind consumers that this dimension is about the first consumption of alternative foods. In this study, we surveyed respondents’ opinions by issuing a questionnaire via the internet, which did not involve interaction or medical-related ethical issues. Therefore, no institutional review board statement was required.

## 3. Research Analysis and Findings

### 3.1. Descriptive Analysis of Demographic Variables

An online questionnaire was distributed to consumers who had experienced innovative alternative foods between October and December 2020. (The questionnaire began by asking if the subject had ever experienced alternative foods). Excepting basic personal data, respondents indicated their level of agreement or disagreement on a 7-point Likert scale ranging from 1 (strongly disagree) to 7. Respondents could access the description of the survey by clicking on the URL link of the questionnaire. During the survey, participants voluntarily answered research questions and had the option to withdraw at any time. The participants agreed to complete the questionnaire on a fully informed and voluntary basis. 

This study collected 519 samples in total. After deleting invalid samples (e.g., logical errors and duplicates), 487 samples remained with an efficiency rate of 93.8%. The ratio of parameter estimate to number of samples (p:n) in the maximum likelihood method should ideally equal 1:10 [59]. There were 19 test variables, 487 valid samples, and a sample size of 1:25.6, exceeding the theoretically recommended value. Therefore, follow-up analyses of the data were conducted. Based on the data provided by the subjects in the valid questionnaire, a statistical analysis was conducted. Table 2 presents the distribution of various demographic variables in the study.

The study sample contained slightly more females than males. In terms of age distribution, the majority of subjects were between the ages of 21 and 30. Furthermore, persons aged 31 to 40 and 41 to 50 constituted a significant portion of the population. There was a relatively even income distribution. Many respondents had received a high school diploma or higher. The majority of respondents were married. It appears that the occupational distribution of respondents was fairly balanced, with a slightly higher proportion of respondents employed in the manufacturing and medical industries. The vast majority of the respondents rated alternative foods as “just so so” or “above”. Previous surveys suggested that women aged 18–29 are more likely to accept alternative foods than men [60]. In this regard, the respondents to the survey in this paper demonstrated the basic qualities of the main consumer groups for alternative foods.

### 3.2. The Reliability Analysis and Exploratory Factor Analysis

For the purposes of enhancing the accuracy of the research results, reliability and item analysis were performed on the questionnaire to eliminate unreliable questions and to ensure the validity and discrimination of the items. Table 3 indicates that all constructs were significantly higher than 0.7 in terms of Cronbach’s alpha, which indicates a high level of reliability. Moreover, the final value fell below the current value if any item of the Cronbach’s α was removed from the construct. This analysis suggested that such items should not be removed. It is important to note that the data are highly reliable, and further study is recommended. 

Next, this study examined the six constructs of the hypothetical model using exploratory factor analysis. As a result, the KMO value for all dimensions exceeded 0.7, and the Bartlett sphericity test did not exceed 0.05, so it could be used for exploratory factor analysis [61,62]. The model extracted six factors with eigenvalues greater than 1; the combined variance explained rate was 81.798%, and a single factor’s explanation was less than 40%; the standard Thompson rule was followed, as there were no common factors explaining the majority of the variances. The results indicated that there were no common methodological variations found in this questionnaire [63]. In addition, the number of factors corresponded to the number of constructs in the default model of the study.

### 3.3. Confirmatory Factor Analysis

#### 3.3.1. Convergent Validity

We used AMOS 22.0 for analysis of the structural equation models. It had been shown in several studies that AMOS is an effective tool for structural equation modeling. Anderson and Gerbing believe that the analysis of information can be divided into two steps [64]. As a first step, we built the measurement model, which uses a maximum likelihood estimation method to measure the parameters of factor loading, reliability, convergent validity, and discriminant validity, in accordance with Hair et al. [65], Nunnally and Bernstein [66], and Fornell and Larcker [67] on convergent validity, and Chin [68] and Hooper [69] et al. on standardized factor loading research. The confirmatory factor testing results are shown in Table 4. Item standardized factor loadings for all variables were greater than 0.5, suggesting that every observed variable contributed to the explanation of its latent variables to a large extent. It should be noted that the combined reliability (CR) was greater than 0.8, which was significantly higher than the standard 0.7. This indicated that the observation variables under each dimension could well explain the dimension. The AVE values in this study all exceeded the standard value of 0.5, indicating a good convergent validity of the scale [65].

The discriminant validity uses the research of Fornell and Larcker [67]. There was robust discriminant validity in the model if the square root of AVE (the average value for each construct) exceeded the correlation coefficient between constructs. The results demonstrated that the diagonal values in this study were greater than the values outside the diagonal, which indicated that all constructs of this study were discriminant valid (see Table 5). Hence, each aspect of this study had high discriminant validity.

#### 3.3.2. Model Fit Test

In accordance with the research of Jackson et al. [70], Kline [71], Schumacker [72], Hu and Bentler [73], and other scholars, this study selected multiple indicators (MLχ^2^, DF, χ^2^/DF, RMSEA, SRMR, TLI, CFI, NFI, GFI, PGFI, PNFI, IFI) for assessing structural model fit. After measuring the parameters in accordance with the model and the hypothesis, Table 6 shows that most of the standard model fit evaluation indicators satisfied both the independent criteria and the combination rule of recommended fitting. The results of the study confirmed that the structural model fit well, and the theoretical framework assumed by the study was consistent with the results.

### 3.4. Path Analysis

According to the path analysis results shown in Table 7, FIQ (b = 0.404, *p* < 0.001) and PV (b = 0.277, *p* < 0.001) were both significantly affected by EXP. These findings indicate that consumers’ expectations about alternative foods are a critical factor in their evaluation of food’s creative design and value. A previous study showed that expectations directly influence consumers’ perceptions of the quality of service and further affect perceived value [74]. In addition, although the measurement of quality of service may differ, similar to the measurement of quality in food innovation, service quality is also a measure of overall quality. The expectation factor is one of the crucial elements in fostering positive perceptions of food innovation quality and perceived value among consumers.

The effect of TRU (b = 0.391, *p* < 0.001) and SAT (b = 303, *p* < 0.001) on CI was significant. The findings indicated that consumers’ continuance intention to accept alternative foods is dependent on both trust and satisfaction. The results confirmed that trust and satisfaction have a significant influence on continuance intention in food marketing [75]. Increasing consumer trust and satisfaction are necessary to ensure a higher level of continuance intention.

The FIQ (b = 0.364, *p* < 0.001) strongly affected SAT. It appears that PV (b = 0.133, *p* = 0.013) also has some influence on SAT. Food innovation quality and food quality may be measured in different ways, but food innovation quality places a greater emphasis on the quality of design that takes an innovative approach to food. Nevertheless, there are implications for quality assessment as well. Thus, this result, supported Konuk [57], indicated that food quality and perceived value are important factors influencing consumer satisfaction with food. In this study, this result established that the influence relationship has been extended to food innovation quality.

TRU is greatly impacted by SAT (b = 0.537, *p* < 0.001). This illustrates that the more trustworthy alternative foods are, the more satisfied the consumer is. According to Al-Ansi et al. [76], a consumer behavior study on halal food demonstrated that trust has a significant impact on consumer satisfaction. As demonstrated by the results of the study, trust is still an important factor in consumer satisfaction with alternative foods.

It is notable that perceived value did not directly affect continuance intention (b = 0.092, *p* > 0.05). Despite the fact that this impact pathway has been demonstrated in other studies [77], it was not valid in the field of food marketing, especially in the study of alternative foods. The findings of this study change perceptions regarding the relationship between perceived value and continuance intention. In conclusion, although perceived value played a significant role in influencing consumers’ opinions about food, it did not necessarily contribute directly to the evaluation of a food’s long-term effects.

### 3.5. Hypothesis Explanation

**Figure 2:**
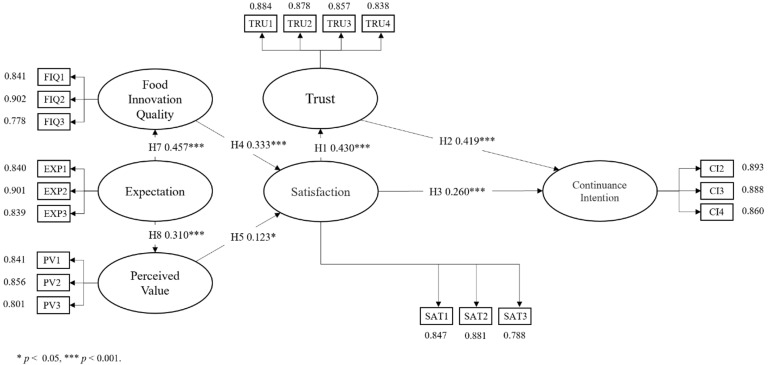
Research structure pattern diagram.

This study was designed to develop a structural equation model to examine the main factors affecting consumer acceptance of artificial foods by those who have consumed them, to test their association, and to suggest possible improvements for government and industry, with reference to consumers, users, and other relevant entities. As can be seen in Figure 2, the relationship between the constructs shows intuitively whether a hypothesis is valid or not.

## 4. Discussion

This study discusses in detail the structural sequence of the research model and the verification results of the structural equation model.

H1, H2, and H3 are valid, suggesting that alternative food has an impact on consumers’ satisfaction, trust, and continuance intention. Thus, consumers’ satisfaction with alternative food experiences has a significant impact on their continued consumption. Similarly, consumer satisfaction affects consumers’ trust in alternative foods, which in turn affects continuance intention. According to the three constructs relationship, satisfaction is positively related to trust, and trust is positively related to continuance intention, which is consistent with the correlation between satisfaction, trust, and continuance intention identified by Lee et al. [78]. These findings support the importance of gaining consumer trust. In the context of this study, consumers might not have been familiar with alternative foods. In order to increase the likelihood of repeat purchases or frequent visits, stores or related practitioners should try to gain the trust of consumers; customer satisfaction is a prerequisite for building trust as well as attracting customers. In the following sections, we will discuss in more detail how to improve consumer satisfaction.

The validity of H4 demonstrates that higher quality of food innovation is one way to increase consumers’ satisfaction. In this study, we divided the quality of food innovation into two parts: the innovation of the substitute recipe itself and the innovation of the dining experience. Higher levels of satisfaction are associated with higher levels of perceived quality of innovation [79]. Since alternative foods are relatively unknown, it is necessary for new innovations to improve their quality to attract different types of consumers, and to try to maintain their consumption. Innovative alternative foods may provide consumers with new experiences and feelings that are different from what they are used to [80]. Consumers may prefer naturalness [35] or familiarity [36]; their individual perception may still result in a relatively high degree of positive perception as a result of the innovative experience. The quality of food innovation, as one of the prerequisite factors affecting consumer satisfaction, may also have positive effects on consumers’ sense of trust and their continuance intentions. Food innovation is also a way to express the innovative quality of food, which was a key aspect of this study.

H5 is valid, whereas H6 is not. This indicates that the value of alternative food perceived by consumers will affect consumer satisfaction, which in turn affects continuance intentions, rather than direct effects. It has always been of particular concern to practitioners how a relatively new type of food (especially insect protein food) will be accepted by consumers. In this study, perceived value was a trade-off between the benefits and costs (or risks) that are recognized by consumers. In the case of new foods, consumers might be more concerned with taste, safety, and price. Additionally, some consumers have already experienced innovative alternative foods that have addressed the above concerns. Multiple perspectives are considered to resolve customer concerns and increase their satisfaction, trust, and continued purchase intention. Accordingly, there is no direct relationship between perceived value and continuance intention. This means there is a process of self-evaluation and transformation within consumers’ experiences, perceptions, acceptances, and decisions, which has implications for practitioners as well.

H7 and H8 are valid. This indicates that consumers’ expectations for alternative food innovations affect consumers’ perceptions of the quality of innovative foods and their perceived value. In previous research, it was found that expectations influence consumer acceptance of food [81]. In addition, they may have learned of alternative foods through curiosity, referrals from friends, advertisements, etc., which may have led to corresponding expectations and subsequent behavior. In terms of consumer perceptions of their experience before and after the purchase of alternative foods, the better experience, food quality, and lower price they expected did meet their expectations. In addition, the model results showed that consumer expectations have the greatest impact on food innovation (0.457). The results also implied that consumers have high expectations for innovative alternative foods and high-quality food, and seek experiences that meet their expectations. 

The comprehensive analysis shows that the four paths of expectation—food innovation quality, satisfaction, -trust, and continuance intention—have the highest coefficients, suggesting that the quality of food and trust in food are indeed very important for consumers’ continuance intention. Despite the high expected-perceived value path coefficient, perceived value has a relatively small effect on satisfaction. In this regard, consumers are more interested in the innovative aspect of alternative foods, which proves that this marketing strategy is indeed appropriate. Although there are numerous channels through which innovation can take place, we believe that for ordinary consumers, innovation should be more about design innovation than technological innovation.

## 5. Theoretical Contribution

This study examined the hypothesis of the relationship path between constructs and the validation of model-based path relationships with respect to the consumers’ intention to continue consuming alternative foods. We attempted to model user behavior by combining the concept of innovative design with the continuance intention of consumers with respect to alternative foods. According to the results of the survey, innovative design is an effective marketing strategy. The validation results of the model confirmed the importance of food innovation quality. In fact, quality is higher than the perceived value that is widely regarded in the study of food marketing as an important factor in promoting consumers’ continuance intentions [82]. This indicates that the quality of food innovation needs to be recognized as a significant construct of alternative food research. Further analysis, and the model constructed around it, are theoretically valuable. As part of the model structure, expectation is an important element for both food innovation quality and perceived value. In other words, expectations regarding alternative foods are the factors that contribute to a positive perception. Previous studies examined extensively the gap between consumers’ expectations and their actual feelings resulting in satisfaction [83]. From a theoretical standpoint, the findings of this paper confirmed once again the importance of satisfying consumer expectations regarding alternative food options. To some extent, the path coefficients and relationships between expectation and satisfaction in the newly proposed model have been updated to take into consideration the type and characteristics of alternative foods. Further, the purpose of this study was not only to provide a model framework from the perspective of innovative design, but also to provide a theoretical framework for future analyses of consumer behavior regarding alternative foods. As a result, future theoretical research will not be affected by an array of research objects. On the one hand, the results of this study were used to update the coefficients and relationships of the theoretical model derived around alternative foods. On the other hand, the study introduced valuable constructs of the model and provided possible directions for further analysis.

## 6. Managerial Implications

Based on a quantitative analysis, this paper proposes some recommendations on how to promote consumers’ actual preferences for and choices of alternative foods. It contributes to the promotion of alternative food as a normalized choice of food. First, innovative food design was a key point of discussion. Considering this, new designs such as how food is prepared, the dining environment, or the marketing strategies have the potential to positively affect consumers’ perceptions of food. Alternative foods, in particular, present a wide array of strangeness and uncertainty [84,85]. Additionally, innovative design can lead to positive emotions. Therefore, to encourage consumers to consume alternative foods, innovative design methods should be initially considered. Second, consumers’ perceptions of food value are equally important. Accordingly, there is a need for moderate promotion and packaging of foods [86,87]. Even though this type of feeling does not directly affect consumers’ continuance intention to consume alternative foods, it does enhance their level of satisfaction. In addition to innovation design, cost-effectiveness promotion can also be incorporated into the systematic development of the marketing strategy. Third, it is essential to pay attention to what consumers are expecting from alternative foods and how these expectations are confirmed by actual tasting. The effectiveness of innovative food design and value promotion for alternative foods is determined by the expectations of consumers. Consequently, setting objectives and achieving desired outcomes are important factors in determining whether a strategy will succeed in encouraging consumers to choose alternative foods. This indicates that good consumer expectations for alternative foods must be established before consumption. Excessive expectations can lead to large differences in expectations and realities, which will in turn decrease satisfaction. It is important that pre-established expectations be selective and restrictive after evaluation. Generally, alternative foods can increase the variety of meat products available on the market and provide consumers with more options [88]. Furthermore, different production processes available on the market may result in a reduction in the numbers of livestock. Consequently, carbon dioxide emissions will be reduced [89]. As a means of rational resource allocation and environmental sustainability, the food industry’s efforts to develop alternative foods are valuable. The purpose of this study was to provide suggestions for a systematic approach to entering the market for alternative foods. Based on the perspective of practical management, this study sets up a way to promote alternative foods in a reasonable and effective manner.

## 7. Conclusions and Suggestions

### 7.1. Conclusions

In this study, an innovative perspective was used to explore and analyze consumers’ continuance intentions towards alternative foods. The model we proposed is based on expectations, food innovation quality, perceived value, satisfaction, trust, and continuance intention, which reflect our understanding of how consumers perceive changes before and after their experience. Insect-based foods are often resisted by consumers, but innovation can also help them become more widely accepted. The empirical findings of this study suggest that consumers’ continuance intentions for alternative foods are influenced by expectation, food innovation quality, perceived value, satisfaction, and trust. In terms of consumer satisfaction, food innovation quality and perceived value are major determinants in the expectation context, and together with trust directly affect continuance intention. Accordingly, it is imperative that relevant practitioners adopt an innovative approach to improving consumer trust in alternative foods, thereby increasing the likelihood of repeat purchases and a higher purchase rate.

### 7.2. Research Limitations and Future Ressarch

Below are some potential directions for future research, in light of the limitations of this study.

1. As alternative foods, especially insect foods, are rarely popular, in order to find enough experienced consumers, we did not limit our research to specific geographical regions. Research could be more in-depth in the future if researchers are able to classify and analyze different consumer groups.
2. The research was based on the perspective of innovation, but the model did not further subdivide the innovation dimension, which was another weakness of this research.
3. As the subjects of this study were consumers who had experienced innovative alternative foods, food safety considerations were not relevant. While food safety was not the focus of this study, food safety should be viewed from the perspective of innovative foods. As a result, future researchers may need to consider the different impacts of food safety on consumers when conducting research relevant to food development or feedback.
4. According to this study, perceived value is defined as consumers’ consideration of cost or whether the food would be worthwhile to consume. The literature we referred to shows that other scholars have divided values into several dimensions, such as nutrition, environment, emotion, etc. Future research may examine the significance of the above dimensions and provide in-depth analyses.

## Figures and Tables

**Table 1:** Definitions of variable operability and reference scales.

Research Variable	Operability Definition	Code	Questions	Reference Scale
Continuance intention	Consumers’ subjective perception of the likelihood of continuing to consume alternative foods in the future.	CI1	I intend to consume alternative foods continuously, not just occasionally.	[18]
		CI2	In the future, I intend to consume alternative foods more frequently.	
		CI3	I’ll recommend alternative foods to my friends.	
Trust	The level of trust consumers have after consuming alternative foods	TR1	Alternative foods are credible in my opinion.	[52,53]
		TR2	Alternative foods are reliable in my opinion.	
		TR3	The alternative food meets my expectations.	
		TR4	Alternative foods can replace traditional foods, in my opinion.	
Satisfaction	Relative relationship between consumers’ actual feelings before and after consuming the alternative food.	SAT1	I am satisfied with the alternative foods.	[52,54]
		SAT2	My payment was better than I expected.	
		SAT3	There is nothing wrong with eating alternative foods.	
Food innovation quality	Consumer perceptions of innovative alternative foods to traditional foods.	FIQ1	I find alternative foods to be innovative.	[54,55]
		FIQ2	In my opinion, alternative foods are fresh and tasty.	
		FIQ3	The quality of the alternative food innovations is high on every visit, in my opinion.	
Perceived value	Consumers’ perceptions of the benefits of alternative foods in comparison to their costs.	PV1	Alternate food is a worthwhile investment.	[56,57]
		PV2	The alternative food is well worth the price that I pay.	
		PV3	Alternative foods are of great value to me.	
Expectation	Consumer experience predicts the availability of alternative foods.	EXP1	I anticipate the alternative food will offer good value for the price I pay.	[58]
		EXP2	In my opinion, alternative foods should be of equal quality to regular foods.	
		EXP3	Alternative foods are expected to be delicious.	

**Table 2:** The basic information of the respondents.

Sample	Category	Number	Percentage (%)
Gender	Male	209	42.92
	Female	278	57.08
Age	Under 20	40	8.21
	21–30 years old	225	46.20
	31–40 years old	95	19.51
	41–50 years old	102	20.94
	Over 51	25	5.13
Monthly Income (RMB)	Under 4000	83	17.04
	4001–6000	183	37.58
	6001–12,000	68	13.96
	12,001–18,000	76	15.61
	18,001–24,000	51	10.47
	Over 24,001	26	5.34
Education Level	Junior high school or lower	93	19.10
	Secondary school or high school	201	41.27
	Undergraduate or college	146	29.98
	Graduate and above	47	9.65
Marital status	Married	393	80.70
	Unmarried	94	19.30
Profession	Manufacturing	135	27.72
	Medical Industry	132	27.10
	Financial Industry	88	18.07
	Design Industry	67	13.76
	Service Industry	65	13.35
How do you feel about alternative foods	Very Good	227	46.61
	Just so so	222	45.59
	Not Good	38	7.80

**Table 3:** The reliability and exploratory factor analysis.

Construct	Item	Cronbach’s α after Deletion	Component					
			1	2	3	4	5	6
Trust α = 0.922	TRU1	0.892	0.827					
	TRU2	0.896	0.826					
	TRU3	0.900	0.848					
	TRU4	0.906	0.797					
Expectation α = 0.894	EXP1	0.861		0.836				
	EXP2	0.821		0.865				
	EXP3	0.862		0.805				
Satisfaction α = 0.878	SAT1	0.816			0.882			
	SAT2	0.795			0.890			
	SAT3	0.866			0.814			
Continuance intention α = 0.913	CI1	0.870				0.819		
	CI2	0.868				0.830		
	CI3	0.886				0.838		
Food Innovation Quality α = 0.876	FIQ1	0.825					0.842	
	FIQ2	0.782					0.864	
	FIQ3	0.865					0.786	
Perceived Value α = 0.871	PV1	0.811						0.881
	PV2	0.805						0.885
	PV3	0.837						0.861
Eigenvalue			3.227	2.489	2.474	2.474	2.447	2.431
Variance explanation after rotation by each factor			16.982	13.102	13.022	13.021	12.877	12.794
Total explained variance%			81.798					
KMO and Bartlett’s Test								
Kaiser-Meyer-Olkin value								0.890
Bartlett’s sphere test					The approximate chi-square			6421.990
					df.			171
					Significance			0.000

**Table 4:** Measurement model.

Construct	Item	Factor Loading	S.E.	t	*p*	CR	AVE
EXP	EXP1	0.839				0.895	0.741
	EXP2	0.893	0.048	23.56	0.000		
	EXP3	0.849	0.045	22.258	0.000		
FIQ	FIQ1	0.841				0.880	0.710
	FIQ2	0.898	0.052	22.67	0.000		
	FIQ3	0.785	0.049	19.692	0.000		
PV	PV1	0.841				0.872	0.695
	PV2	0.858	0.055	20.357	0.000		
	PV3	0.801	0.048	19.315	0.000		
SAT	SAT1	0.854				0.880	0.710
	SAT2	0.886	0.047	22.176	0.000		
	SAT3	0.784	0.045	19.722	0.000		
TRU	TRU1	0.887				0.922	0.748
	TRU2	0.875	0.035	26.884	0.000		
	TRU3	0.855	0.037	25.678	0.000		
	TRU4	0.842	0.037	24.961	0.000		
CI	CI1	0.894				0.913	0.777
	CI2	0.892	0.037	27.496	0.000		
	CI3	0.858	0.037	25.767	0.000		

**Table 5:** Discriminant validity for the measurement model.

	EXP	FIQ	PV	SAT	TRU	CI
**EXP**	**0.860**					
**FIQ**	0.450	**0.842**				
**PV**	0.299	0.239	**0.833**			
**SAT**	0.297	0.316	0.176	**0.842**		
**TRU**	0.509	0.584	0.405	0.549	**0.864**	
**CI**	0.625	0.480	0.433	0.308	0.228	**0.881**

**Table 6:** Evaluation results.

Indicators	Norm	Results	Judgment
ML chi-square (MLχ^2^)	The small the better	183.943	
Degrees of Freedom (DF)	The large the better	137	
Normed Chi-square (χ^2^/DF)	1 < χ^2^/DF < 5	1.343	Yes
Root Mean Square Error Approximation (RMSEA)	<0.08	0.027	Yes
Standardized Root Mean Square Residual (SRMR)	<0.08	0.030	Yes
Tucker-Lewis Index (TLI)	>0.9	0.991	Yes
Comparative Fit Index (CFI)	>0.9	0.993	Yes
Normative Fit Index (NFI)	>0.9	0.972	Yes
Goodness of Fit Index (GFI)	>0.8	0.962	Yes
Parsimony Goodness of Fit Index (PGFI)	>0.5	0.694	Yes
Parsimony Normed Fit Index (PNFI)	>0.5	0.779	Yes
Incremental Fit Index (IFI)	>0.9	0.993	Yes

**Table 7:** Regression coefficient.

Hypothesis	DV	IV	Unstd	S.E.	Unstd/S.E.	*p*-Value	Std.	Results
H1	TRU	SAT	0.537	0.061	8.843	0.000	0.430	Support
H2	CI	TRU	0.391	0.045	8.647	0.000	0.419	Support
H3	CI	SAT	0.303	0.057	5.282	0.000	0.260	Support
H4	SAT	FIQ	0.364	0.055	6.600	0.000	0.333	Support
H5	SAT	PV	0.133	0.054	2.484	0.013	0.123	Support
H6	CI	PV	0.092	0.055	1.692	0.091	0.073	nonsupport
H7	FIQ	EXP	0.404	0.044	9.178	0.000	0.457	Support
H8	PV	EXP	0.277	0.045	6.134	0.000	0.310	Support

## Data Availability

No new data were created or analyzed in this study. Data sharing is not applicable to this article.
